# Cognitive science theory-driven pharmacology elucidates the neurobiological basis of perception-motor integration

**DOI:** 10.1038/s42003-022-03864-1

**Published:** 2022-09-06

**Authors:** Elena Eggert, Astrid Prochnow, Veit Roessner, Christian Frings, Alexander Münchau, Moritz Mückschel, Christian Beste

**Affiliations:** 1 Cognitive Neurophysiology, Department of Child and Adolescent Psychiatry, Faculty of Medicine, TU Dresden, Dresden, Germany; 2 University Neuropsychology Center, Faculty of Medicine, TU Dresden, Dresden, Germany; 3 Cognitive Psychology, Institute of Psychology, University of Trier, Trier, Germany; 4 Institute of Systems Motor Science, University of Lübeck, Lübeck, Germany

**Keywords:** Cognitive control, Human behaviour

## Abstract

An efficient integration of sensory and motor processes is crucial to goal-directed behavior. Despite this high relevance, and although cognitive theories provide clear conceptual frameworks, the neurobiological basis of these processes remains insufficiently understood. In a double-blind, randomized placebo-controlled pharmacological study, we examine the relevance of catecholamines for perception-motor integration processes. Using EEG data, we perform an in-depth analysis of the underlying neurophysiological mechanisms, focusing on sensorimotor integration processes during response inhibition. We show that the catecholaminergic system affects sensorimotor integration during response inhibition by modulating the stability of the representational content. Importantly, catecholamine levels do not affect the stability of all aspects of information processing during sensorimotor integration, but rather—as suggested by cognitive theory—of specific codes in the neurophysiological signal. Particularly fronto-parietal cortical regions are associated with the identified mechanisms. The study shows how cognitive science theory-driven pharmacology can shed light on the neurobiological basis of perception-motor integration and how catecholamines affect specific information codes relevant to cognitive control.

## Abstract (web summary)

An evaluation of predictions from the proposed BRAC framework of sensorimotor integration suggests that the catecholaminergic system might impact sensorimotor integration during response inhibition by modulating the stability of the representational content.

## Introduction

An efficient integration of sensory and motor processes is crucial to goal-directed behavior, which relies on both the execution and inhibition of responses^1^. Several lines of evidence suggest that sensory processes have a strong impact on successful response inhibition performance^2–4^. However, the neurobiological basis of these fundamental processes continues to be the subject of ample neuroscientific research which requires further investigation^5^. In the current study, which was inspired by recent theorizing in cognitive science, we performed a neuropharmacological investigation regarding the importance of the catecholaminergic system for sensorimotor integration and the underlying neurophysiological processes during response inhibition. Two cognitive science frameworks which describe the mechanisms of sensorimotor integration are the Theory of Event Coding (TEC)^6^ and the more recent Binding and Retrieval in Action Control (BRAC) framework^7^. While the TEC mostly focuses on the event file coding process and the associated binding effects, the Binding and Retrieval in Action Control framework extends the TEC, emphasizing the sequential prime-probe structure commonly employed in associated experimental task paradigms while aiming to provide an overarching, paradigm-independent approach^7^. The Binding and Retrieval in Action Control framework specifies the operations with an event file and describes how event files are managed. Both theories postulate the idea of common coding^8^, namely the assumption that representations of features detailing stimuli (i.e., the stimulus’ color, form, orientation, shape, brightness etc.) are connected to each other and represented together with features detailing the response or motor actions prompted by the incoming information (i.e., the response effector like hand, arm, finger, and movement characteristics including velocity, force etc.). Features are integrated or bound into so-called event files^9^. Event files can be thought of as (episodic) memory traces containing the information regarding a stimulus, an associated response as well as the respective stimulus-response relationship, all of which is connected and embedded in a network-like structure^10^. This structure of an event file entails that once a stimulus is presented, the entire network—including the corresponding response-related information—becomes activated. This activation pattern may cause problems whenever ambiguous stimuli (e.g., stimuli that display features triggering two opposite classes of responses) are presented. If, for example, a stimulus is presented which includes some features prompting a motor response along with features triggering the inhibition of a response, the likelihood is high that a response will be executed even though it is supposed to be inhibited^11^. This effect has been shown using a Go/Nogo task modified on the basis of the TEC^11,12^, which was also the task administered in the current study. The stimuli in this task consisted of words and letters in different font colors. On the one hand, there were non-overlapping Go and Nogo conditions, which differed completely with respect to the words and font colors used. On the other hand, there were Go and Nogo conditions in which the words and font colors overlapped. Here, for example, the same word might constitute a Go stimulus in one font color, but a Nogo stimulus in another font color. Previous studies using the task have demonstrated longer reaction times and higher error rates in these overlapping conditions^11,12^. Such declines in performance are referred to as partial repetition costs^9,13^. The strength of the represented event file bindings has been assumed to impact such effects^9,14,15^. Therefore, a neuropsychopharmacological modulation of the strength of event file bindings and representations can be assumed to affect the integration of sensory and motor processes.

The stability of memory representations^16,17^ strongly depends on the dopaminergic^18–20^ and norepinephrinergic systems^21^. Therefore, both neurotransmitter systems should be modulated concomitantly to produce strong effects, which can be achieved by using methylphenidate (MPH). MPH is a combined dopamine and norepinephrine transporter blocker that increases both dopamine and norepinephrine concentrations in prefrontal and cortico-striatal circuits^22–26^. Specifically, on the cellular level, MPH as an indirect agonist inhibits the reuptake of dopamine and norepinephrine by binding to the respective neurotransmitter transporter and facilitates the release of both neurotransmitters into the synaptic cleft, thereby increasing catecholamine levels^27,28^. One conceivable effect to ensue after MPH intake consists in an impairment of response inhibition performance when stimuli triggering response inhibition also display perceptual features which indicate to execute a response. Due to the increased strength of event file representations after MPH intake, it might thus be particularly difficult to reconfigure the event files efficiently when necessary. However, recent evidence related to the Metacontrol State Model^29,30^ has shown a lower proneness to interference effects when there is a high cognitive stability^31^. Since interference and conflicts can compromise response inhibition^32,33^, it is also possible that perception-motor integration during response inhibition improves after increasing catecholaminergic neural transmission.

Regardless of the direction of the outcome, pharmacological effects should be ascribable to specific processes at the neurophysiological level. If it is specifically the representational strength of an event file that is central to understanding the effects of catecholaminergic modulation on the integration of sensory and motor processes during response inhibition, a change in the stability of information contained in neurophysiological processes should be reflected in form of MPH effects. Using electroencephalography (EEG) data, these processes can be examined by performing multivariate pattern analysis (MVPA)^34–37^ and temporal generalization MVPA in particular^37^. In comparison with classical univariate tests, MVPA demonstrates a high sensitivity to the identification of multivariate dependencies between activity sources in different locations in the brain^38^. Examining the dynamics of the observed neural activity patterns, MVPA uses a classifier to detect systematic differences in the EEG signal between two conditions. The training of the classifier to every channel allows for a less focal approach, taking into account distributed encoded information. Further, MVPA can estimate whether it is possible to generalize an obtained classification to other time points: Temporal generalization MVPA can identify when a specific aspect of information is encoded into brain activity and can provide insight regarding the changes of representational content over time^35,39^. The time courses of the EEG signal in two conditions are contrasted. If a generalization over time is found, i.e., if several consecutive time points in one condition can be predicted from the EEG signal in the other condition, this can be interpreted as the temporal stability of mental representations^37^. Therefore, it is possible to determine when and for how long information is represented in the neural code by using temporal generalization MVPA. In the context of event file research, the investigation of the temporal stability of representations is particularly relevant. We expect that the temporal stability of event file representations is higher after MPH intake and that this effect is particularly evident when stimuli which trigger response inhibition also include perceptual features which indicate to execute a response. Therefore, the main focus of the current study will be to contrast the neurophysiological signals in a session with MPH with the signals in a session with a placebo in the Nogo condition with stimuli that share features with Go stimuli.

However, recent research has suggested that codes reflecting the integration of perception and action in event files are only contained in specific fractions of the neurophysiological signal^40–42^. Therefore, we applied a temporal signal decomposition (i.e., residue iteration decomposition, RIDE^43,44^) prior to the MVPA. Using RIDE, the EEG signal is decomposed into three clusters (S-cluster, C-cluster, and R-cluster), each of which has the same data structure as the original EEG data^44^. The clusters are derived from the original signal using time markers in the EEG; for example, the S-cluster contains signals that are related to the time point of the stimulus onset. Therefore, each cluster represents a different cognitive processing stage: the S-cluster is associated with stimulus-related perception and attention processes, the R-cluster, which is related to the time point of the response, reflects motor response execution, and the C-cluster, which contains signals that are neither directly linked to the stimulus onset nor to the response, is related to processes of response selection and thus stimulus-response associations^44,45^. This clustering reflects conceptual assumptions of the TEC, as this theory assumes a stimulus-related object file (S-cluster) as well as a response-related action file (R-cluster) are integrated in the event file (C-cluster)^15,41,42,46^. The impact of the catecholaminergic system on event file processes should be particularly evident in the C-cluster, since catecholaminergic modulation has been shown to affect the stability of stimulus-response associations^16,17^ and stimulus-response translational processes have been found to be reflected by the C-cluster^15,40–42^. Since no response is required in the Nogo trials of a Go/Nogo paradigm, only the S-cluster and the C-cluster are derived in the current study^47^.

In order to examine which functional neuroanatomical structures are associated with modulations of the stability of the representational content in specific fractions of neurophysiological activity, source localization was performed using sLORETA (standard low resolution brain electromagnetic tomography^48^). Brain regions subserving inhibitory processes and event file dynamics largely overlap: Parietal areas are thought to be related to inhibitory processes in the event of complex stimulus input that is hard to categorize^49–53^. The current study utilizes such input to examine perception-motor integration during inhibitory control. Moreover, parietal areas are involved in the updating of mental representations, which is particularly important for the reconfiguration of event files^40,54,55^. Furthermore, orbitofrontal and inferior frontal areas, such as the right inferior prefrontal cortex, are related to the inhibitory control network^1,56,57^ and are known to be modulated by MPH administration^58^. Importantly, medial and superior frontal areas are crucial for inhibitory processes^1,59^ and are associated with stimulus-response integration processes^15,42^. Previous findings have suggested activity in these areas can be modulated by MPH, as well^60^. Taken together, we hypothesize that modulations of memory representations (examined via MVPA) by means of a manipulation of the catecholaminergic system (via MPH administration) are most evident in the C-cluster and can be associated with the superior and medial frontal, orbitofrontal, lateral inferior frontal and parietal areas. To this end, we administered the Go/Nogo paradigm described above which included Go and Nogo trials that could both be either overlapping or non-overlapping. In the overlapping trials, the features of the stimuli in the Go condition were partly the same as the features of the stimuli in the Nogo condition. In the non-overlapping condition, there were no shared features between the Go and Nogo stimuli. In a randomized, double-blind cross-over design, all participants took part in two sessions, receiving the MPH compound in one appointment and the placebo substance in the other. The obtained EEG data were analysed on the behavioral as well as on the neurophysiological level.

## Results

### Behavioral data: MPH modulates inhibitory control when demands on perception-motor integration are high

**Fig. 1:**
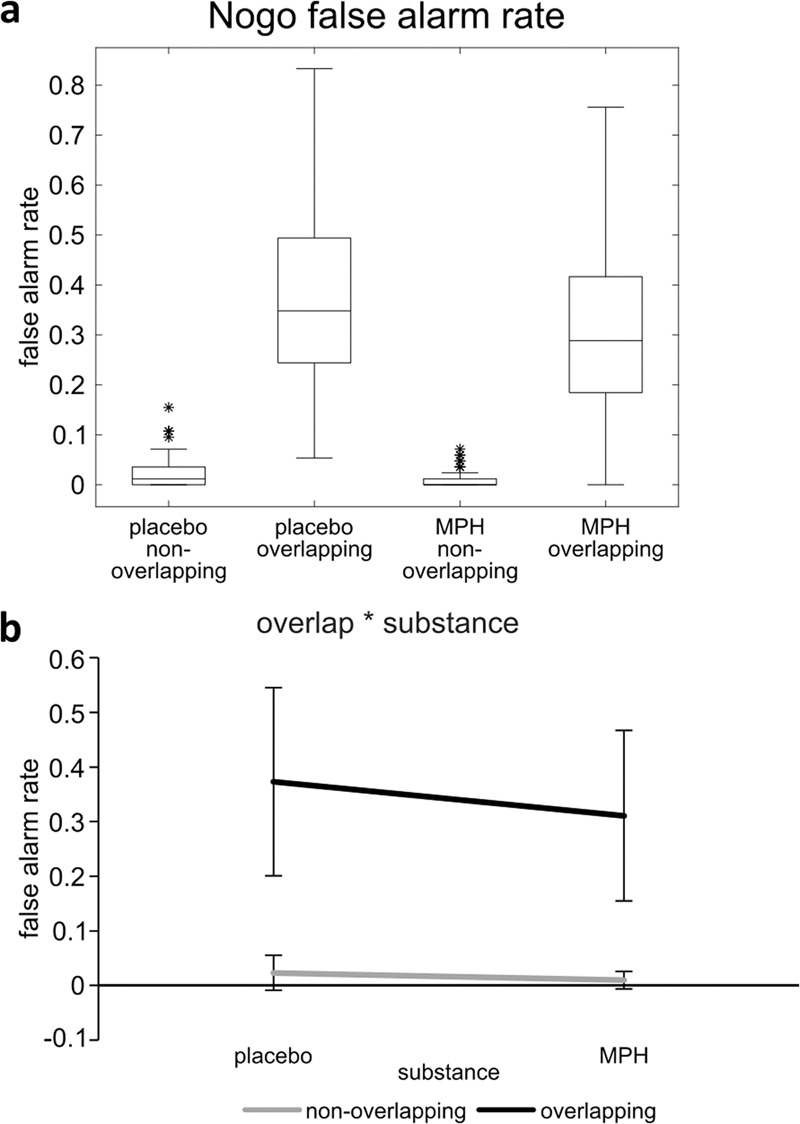
False alarm rates of the non-overlapping and overlapping Nogo conditions in the placebo and MPH session. **a** Boxplots of the distribution of the false alarm rates of the non-overlapping and overlapping Nogo conditions in the placebo and MPH session of *N* = 78 participants. The sample median is shown as the line inside the box, the lower and upper quartiles are shown as the bottom and top edges of the box, the ends of the whiskers denote the non-outlier minimum and maximum, respectively, asterisks denote outliers. **b** Illustration of the significant interaction of the factors Overlap and Substance (same data as figure part **a**). Error bars represent standard deviations.

The distribution of the behavioral data in the Nogo trials is displayed in Fig. 1a, an illustration of the interaction of Overlap*Substance can be seen in Fig. 1b. The proportion of Nogo trials with an erroneously given response in a particular condition out of all Nogo trials in that condition constituted the Nogo false alarm rate.

A mixed-effects ANOVA with the within-subject factors Overlap (non-overlapping vs. overlapping condition) and Substance (placebo vs. MPH session) and the between-subjects factor Group (Placebo-first vs. MPH-first) was conducted. The analysis revealed main effects of the factors Substance (F(1,76) = 38.70, *p* < 0.001, η_p_² = 0.337) and Overlap (F(1,76) = 396.51, *p* < 0.001, η_p_² = 0.839), with higher false alarm rates in the placebo session (0.20 ± 0.10) than in the MPH session (0.16 ± 0.08) and higher false alarm rates in the overlapping (0.34 ± 0.16) than in the non-overlapping condition (0.02 ± 0.02). Further, the interaction of the within-subject factors Substance*Overlap was significant (F(1,76) = 23.20, *p* < 0.001, η_p_² = 0.234). Prior to any post-hoc tests, Kolmogorov-Smirnov-tests were conducted for all post-hoc variables to test for normality. Wilcoxon-tests revealed significant binding effects in the placebo session (non-overlapping: 0.02 ± 0.03, D(78) = 0.27, *p* < 0.001; overlapping: 0.37 ± 0.17, D(78) = 0.08, *p* = 0.200; Z = −7.67, *p* < 0.001) as well as in the MPH session (non-overlapping: 0.01 ± 0.02, D(78) = 0.33, *p* < 0.001; overlapping: 0.31 ± 0.16, D(78) = 0.07, *p* = 0.200; Z = −7.62, *p* < 0.001), with a larger binding effect in the placebo session (0.35 ± 0.15, D(78) = 0.06, *p* = 0.200) than in the MPH session (0.30 ± 0.15, D(78) = 0.08, *p* = 0.200; t(77) = 4.83, *p* < .001). The effect of substance was larger in the overlapping (−0.06 ± 0.09, D(78) = 0.09, *p* = 0.200) than in the non-overlapping condition (−0.01 ± 0.03, D(78) = 0.26, *p* < 0.001; Z = −4.18, *p* < 0.001). Bayesian statistics revealed a value of BF_10_ = 2.35 × 10^12^ for the interaction of Substance*Overlap, indicating very strong evidence for the alternative hypothesis relative to the null hypothesis. None of the main effects and interactions including the factor Group reached significance (all F ≤ 1.72, all *p* ≥ 0.193). Therefore, the analyses of the neurophysiological data were conducted averaged across Group. Since particularly the Nogo trials are relevant to the research question examined, the results for the Go hit rate and the Go reaction times are given in the Supplementary material (Supplementary Note 1 and Supplementary Figs. 1 and 2). Taken together, the behavioral results showed higher false alarm rates in the placebo session than in the MPH session and higher false alarm rates in the overlapping than in the non-overlapping condition. Furthermore, the findings demonstrated an interaction effect between Substance and Overlap, revealing a stronger binding effect in the placebo session than in the MPH session.

### Neurophysiological data: MPH modulates the stability of the representational content of response selection codes

The EEG data were decomposed into two clusters using RIDE. Subsequently, MVPA was performed on the undecomposed as well as with the decomposed RIDE data. Furthermore, the associated neuroanatomical regions were investigated using source localization (sLORETA).

**Fig. 2:**
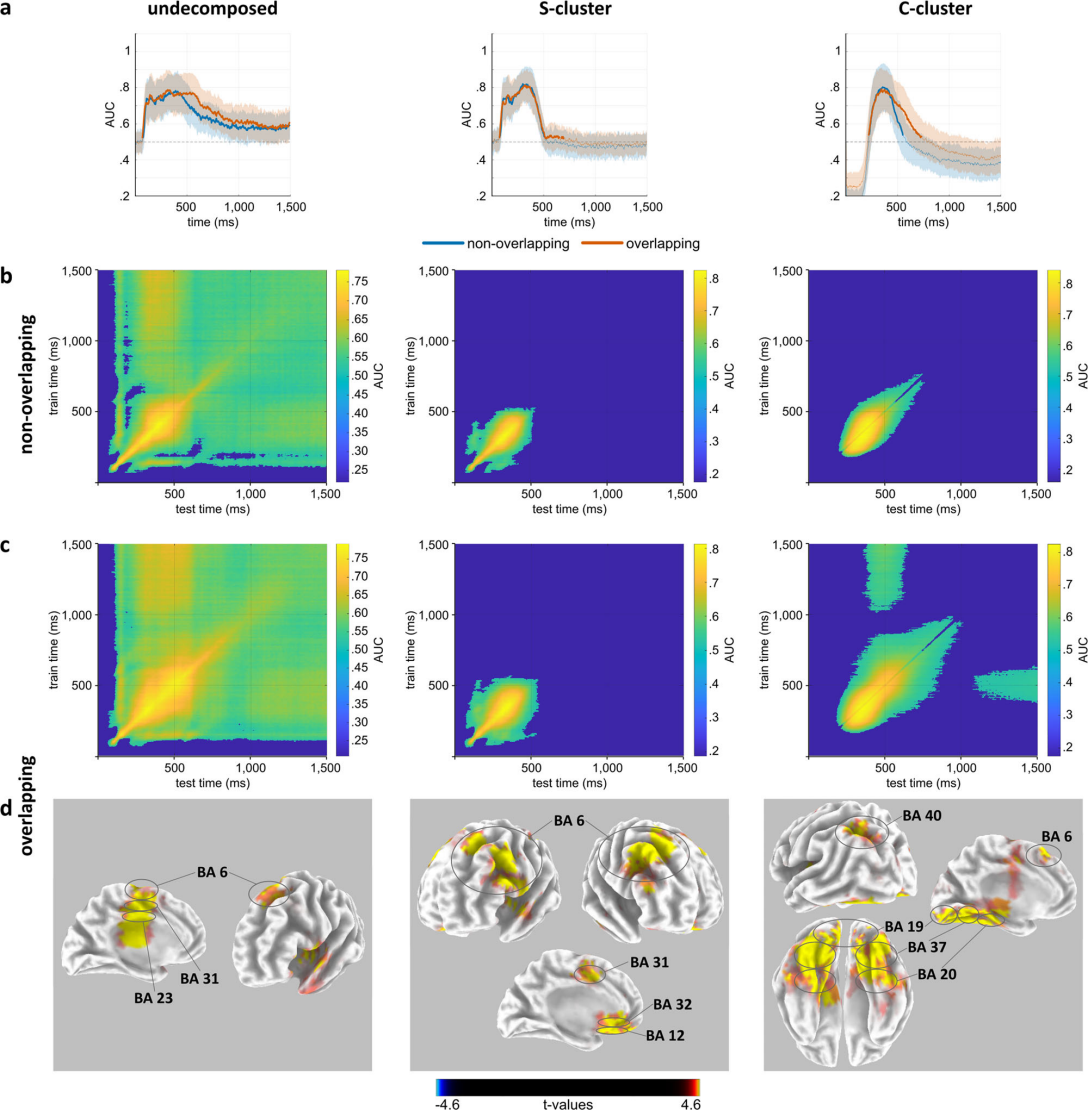
Results of the MVPA comparing the placebo session and the MPH session in Nogo trials. Results of the MVPA comparing the placebo session and the MPH session in Nogo trials in the undecomposed EEG data, in the RIDE S-cluster and in the RIDE C-cluster of *N* = 78 participants. Figure part **a** shows the AUC for the diagonal activity in the non-overlapping condition (blue line) and the overlapping condition (orange line); thick lines indicate significant above-chance classification, the shading around the line represents the standard deviation of the AUC across the sample. Figure part **b** shows the temporal generalization matrices for the non-overlapping condition, whereas figure part **c** shows the temporal generalization matrices for the overlapping condition; the color scaling reflects the level of classification accuracy as indicated by the AUC. **d** shows the results of the sLORETA in the time window of significant AUC in the overlapping condition. Color indicates t-values.

The MVPA was applied to the undecomposed as well as to the RIDE-decomposed data in the time window of 0 to 1500 ms relative to the stimulus onset using the MVPA-light toolbox^38^. First, the classes of MPH and placebo were compared in the overlapping condition and non-overlapping condition. To this end, a binary classification analysis was run to determine time points with values that differed between the MPH and the placebo session. Subsequently, in order to examine the stability of the representations over time, temporal generalization matrices were computed. Further, a source localization using sLORETA^48^ was conducted for the time periods of above-chance classification performance in the overlapping condition. The alpha value was set to 0.05. Figure 2 displays the results of the binary classification in the overlapping and the non-overlapping condition (Fig. 2a), the results of the temporal generalization MVPA in the non-overlapping (Fig. 2b) and the overlapping condition (Fig. 2c) as well as the results of the sLORETA for the overlapping condition (Fig. 2d).

In the undecomposed data (Fig. 2 left panel), the analysis revealed significant differences (*p* < 0.05) between the placebo and MPH session in both the overlapping and the non-overlapping condition. In the non-overlapping condition, an above-chance classification performance with a classification accuracy ranging between 0.52 and 0.78 (mean classification accuracy: 0.64) was observed in the time period from 74 to 1500 ms after stimulus onset. The temporal generalization had a duration of about 1330 ms on average. In the overlapping condition, an above-chance classification performance with a classification accuracy ranging between 0.52 and 0.79 (mean classification accuracy: 0.67) was observed in the time period from 70 to 1500 ms after stimulus onset. The temporal generalization had a duration of about 1350 ms on average. As can be seen, the range and duration of above-chance classification accuracy did not differ substantially between conditions. In the time period of above-chance classification performance, sLORETA revealed modulations in the superior frontal gyrus (BA 6) and the anterior cingulate cortex (BA 23, BA 31) in the overlapping condition.

In the RIDE S-cluster (Fig. 2 center panel), the analysis revealed significant differences (*p* < 0.05) between the placebo and MPH session in both the overlapping and the non-overlapping condition. In the non-overlapping condition, an above-chance classification performance with a classification accuracy ranging between 0.52 and 0.82 (mean classification accuracy: 0.73) was observed in the time period from 70 to 508 ms after stimulus onset. The temporal generalization around the diagonal had a duration of about 275 ms on average. In the overlapping condition, an above-chance classification performance with a classification accuracy ranging between 0.52 and 0.81 (mean classification accuracy: 0.67) was observed in the time period from 70 to 695 ms after stimulus onset with a short break from 656 to 668 ms after stimulus onset. The temporal generalization around the diagonal had a duration of about 340 ms on average. As can be seen, the duration of above-chance classification accuracy was larger in the overlapping than in the non-overlapping condition. In the time period of above-chance classification performance, sLORETA revealed modulations in the superior frontal gyrus (BA 6), the anterior cingulate cortex (BA 31) and the orbitofrontal cortex (BA 12, BA 32) in the overlapping condition.

Similarly, the analysis of the RIDE C-cluster data showed significant differences (*p* < 0.05) for both the overlapping condition and the non-overlapping condition between placebo and MPH session (Fig. 2 right panel). With a classification accuracy of 0.54 to 0.80 (mean classification accuracy: 0.71), an above-chance classification performance was revealed in the time window from 227 to 555 ms after stimulus onset in the non-overlapping condition. The temporal generalization around the diagonal had a duration of about 335 ms on average. In the overlapping condition, an above-chance classification performance with a classification accuracy of 0.52 to 0.79 (mean classification accuracy: 0.68) was shown in the time window from 219 to 734 ms after stimulus onset. The temporal generalization around the diagonal had a duration of about 525 ms on average. As can be seen, the duration of above-chance classification accuracy was larger in the overlapping than in the non-overlapping condition. In the time period of above-chance classification performance, sLORETA revealed modulations in the superior parietal cortex (BA 40), the superior frontal gyrus (BA 6) and parts of the temporal and occipital cortex (BA 19, BA 20, BA 37) in the overlapping condition. In the C-cluster, a classification performance below chance could be observed before the chosen time window (150 to 800 ms relative to stimulus onset), indicating the lack of a valid signal in the first 150 ms after the presentation of the stimulus in the C-cluster.

Taken together, the findings demonstrate a successful classification performance for both the S-cluster and the C-cluster. Importantly, the classification performance was more pronounced in the overlapping than in the non-overlapping condition. Within the overlapping condition it was even more pronounced in the RIDE C-cluster data than in the RIDE S-cluster data. A more restrictive alpha value of 0.001 did not meaningfully change the results (see Supplementary Figs. 4 and 5). In order to provide a complete analysis, an additional MVPA comparing the non-overlapping and the overlapping conditions in the placebo and the MPH session was conducted. Since this comparison is not part of the main research question on the effects of MPH intake, the results of these analyses can be found in the Supplementary material (Supplementary Note 2 and Supplementary Fig. 3).

### Neurophysiological data: No consistent effects of MPH on neurophysiological processes unrelated to representational content

**Fig. 3:**
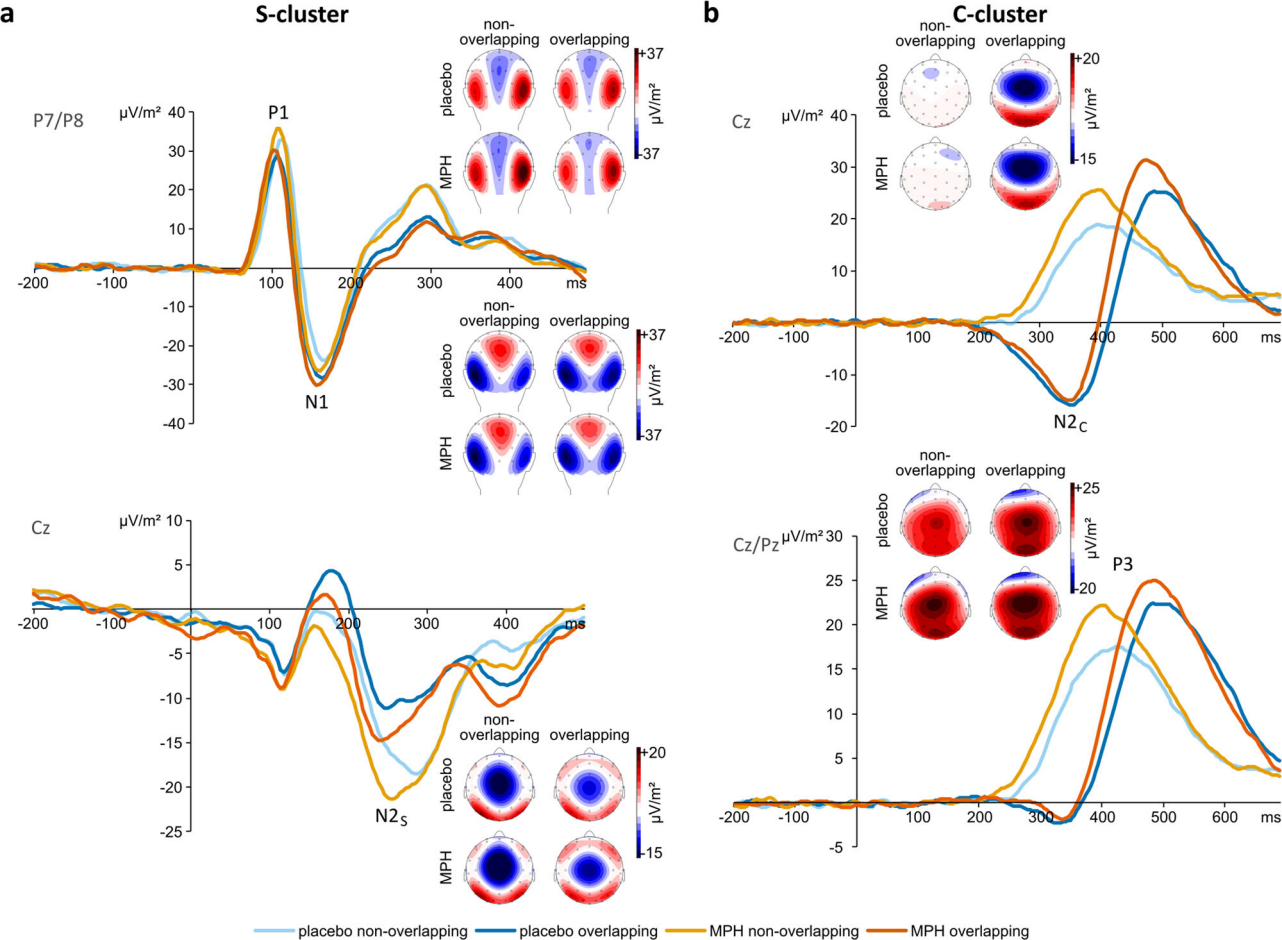
Event-related components in the RIDE S- and C-cluster. RIDE data of *N* = 78 participants in **a** the RIDE S-cluster (P1 and N1 pooled across electrodes P7 and P8, N2_S_ on electrode Cz), and **b** the RIDE C-cluster (N2_C_ on electrode Cz, P3 pooled across electrodes Cz and Pz). The lines for the placebo session are shown in blue, the lines for the MPH session are shown in orange. The non-overlapping condition is shown in a lighter color than the overlapping condition. Time point 0 denotes the time point of the stimulus onset. Topographic plots show the distribution of the potentials at the peak of the respective component. Positive potentials are shown in red, negative potentials are shown in blue, scaling is given in μV/m².

The analysis outlined above shows that MPH affects the stability of the representational content in specific fractions of the neurophysiological signal. To examine whether only these effects or also other neurophysiological processes unrelated to the stability of the representational content are affected, we also examined standard time-domain properties of the EEG signal. We examined the RIDE-decomposed time domain data. For all post-hoc tests, Kolmogorov-Smirnov tests were performed. In case of a lack of normality of the data, Wilcoxon tests were calculated for the respective post-hoc comparison. A Bayesian analysis was carried out for each component for the interaction between Substance and Overlap. Figure 3 displays the time courses of activity at certain electrodes and the topographic plots during the analyzed time windows in the RIDE S-cluster (Fig. 3a) and the RIDE C-cluster (Fig. 3b). Table 1 shows a summary of the results pertaining to the interaction effect Substance*Overlap of the RIDE-ERPs.

**Table 1:** Interaction effect Substance*Overlap.

Cluster	RIDE-ERP	Electrode(s)	F-value	p-value	BF_10_
S-cluster	RIDE-P1	P7 vs. P8	7.06	0.010**	1880.04
	RIDE-N1	P7 vs. P8	1.23	0.270	0.42
	RIDE-N2_S_	Cz	0.58	0.448	0.16
C-cluster	RIDE-N2_C_	Cz	0.12	0.727	0.08
	RIDE-P3	Cz vs. Pz	0.84	0.362	0.23

Overview of the interaction effect Substance*Overlap of the RIDE-ERPs with the corresponding F-values, *p*-values and BF_10_-values, as calculated with individual repeated-measures ANOVAs. **p ≤ 0.01, *p ≤ 0.05.

The analysis of the neurophysiological data for the RIDE-P1 (i.e., the P1 after RIDE) was carried out in a repeated-measures ANOVA with the within-subject factors Electrode (P7 vs. P8), Substance (placebo vs. MPH) and Overlap (overlapping vs. non-overlapping). The data showed a main effect of Electrode (F(1,77) = 18.88, *p* < 0.001, η_p_² = 0.197), with a higher amplitude for the P8 (33.37 ± 20.83 µV/m²) than for the P7 (23.23 ± 15.79 µV/m²). Furthermore, the analysis showed a main effect of Substance (F(177) = 10.93, *p* = 0.001, η_p_² = 0.124): The amplitude was higher in the MPH session (30.43 ± 15.97 µV/m²) than in the placebo session (26.16 ± 16.77 µV/m²). Moreover, there was a main effect of Overlap (F(1,77) = 7.37, *p* = 0.008, η_p_² = 0.087), with a higher amplitude in non-overlapping condition (29.23 ± 16.52 µV/m²) than in overlapping condition (27.36 ± 14.72 µV/m²). Importantly, the analysis showed an interaction effect between Substance and Overlap (F(177) = 7.06, *p* = 0.010, η_p_² = 0.084): In the placebo session, there was no difference between the overlapping condition (25.90 ± 16.57 µV/m², D(78) = 0.10, *p* = 0.038) and the non-overlapping condition (26.42 ± 17.80 µV/m², D(78) = 0.11, *p* = 0.028; Z = −0.96, *p* = 0.335). In the MPH session, on the other hand, there was a higher amplitude in the non-overlapping condition (32.04 ± 17.65 µV/m², D(78) = 0.06, *p* = 0.200) compared to the overlapping condition (28.83 ± 15.05 µV/m², D(78) = 0.05, *p* = 0.200; t(77) = −3.81, *p* < 0.001). The effect of substance was evident in both the non-overlapping (Z = −4.25, *p* < 0.001) and the overlapping condition (Z = −2.94, *p* = 0.003), but it was larger in the non-overlapping condition (non-overlapping: 5.62 ± 12.87 µV/m², D(78) = 0.12, *p* = 0.010; overlapping: 2.93 ± 11.63 µV/m², D(78) = 0.12, *p* = 0.005; Z = −2.37, *p* = 0.018). Bayesian statistics revealed a value of BF_10_ = 1880.04 for the interaction of Substance*Overlap, indicating very strong evidence for the null hypothesis relative to the alternative hypothesis. No other effects were shown to be significant (all F ≤ 3.65, all *p* ≥ 0.060). In order to further investigate the relation of the RIDE-P1 and the behavioral data, a correlational analysis was conducted between the false alarm rates and the RIDE-P1 amplitude in the four conditions as well as between the differences between conditions, i.e., the binding effects and the substance effects. The following significant correlations were obtained: a positive correlation between the behavioral substance effect in the non-overlapping condition and the RIDE-P1 binding effect in the Placebo session (r(76) = 0.23, *p* = 0.047); a negative correlation between the behavioral binding effect in the MPH session and the RIDE-P1 binding effect in the placebo session (r(76) = −0.30, *p* = 0.009); a positive correlation between the false alarm rate in the non-overlapping condition in the MPH session and the RIDE-P1 binding effect in the MPH session (r(76) = 0.26, *p* = 0.020); a negative correlation between the false alarm rate in the overlapping condition in the MPH session and the RIDE-P1 binding effect in the placebo session (r(76) = −0.27, p = 0.016). None of the other correlations between the behavioral false alarm rates and RIDE-P1 amplitudes was significant (|*r* | < 0.19, *p* > 0.094).

For the RIDE-N1 in the S-cluster, a repeated-measures ANOVA with the factors Electrode (P7 vs. P8), Substance (placebo vs. MPH) and Overlap (overlapping vs. non-overlapping) revealed a main effect of Electrode (F(177) = 8.01, *p* = 0.006, η_p_² = 0.094), with a larger (i.e., more negative) amplitude for the P7 (−29.04 ± 24.30 µV/m²) than for the P8 (−22.25 ± 24.00 µV/m²), as well as a main effect of Overlap (F(1,77) = 32.39, *p* < .001, η_p_² = 0.296), with a larger (i.e., more negative) amplitude in the overlapping condition (−27.99 ± 22.67 µV/m²) than in the non-overlapping condition (−23.31 ± 21.31 µV/m²). Finally, the analysis showed an interaction effect between Electrode and Overlap (F(1,77) = 83.12, *p* < 0.001, η_p_² = 0.519). Post-hoc tests established that there was no significant difference between overlapping condition (−28.74 ± 24.98 µV/m², D(78) = 0.08, *p* = 0.200) and non-overlapping condition (−29.35 ± 24.20 µV/m², D(78) = 0.08, *p* = 0.200) at the P7 (t(77) = 0.71, *p* = 0.481), while there was a significantly larger (i.e., more negative) amplitude in the overlapping condition (−27.24 ± 25.08 µV/m², D(78) = 0.10, *p* = 0.075) than in the non-overlapping condition (−17.27 ± 23.94 µV/m², D(78) = 0.09, *p* = 0.192) at the P8 (t(77) = −8.78, *p* < 0.001). All other effects failed to reach significance (all F ≤ 2.06, all *p* ≥ 0.155). Bayesian statistics revealed a value of BF_10_ = 0.42 for the interaction of Substance*Overlap, indicating weak evidence for the null hypothesis relative to the alternative hypothesis.

For the RIDE-N2_S_ at the Cz in the S-cluster, a repeated-measures ANOVA with the factors Substance (placebo vs. MPH) and Overlap (overlapping vs. non-overlapping) yielded a main effect of Substance (F(1,77) = 11.00, *p* = 0.001, η_p_² = 0.125), with a larger (i.e., more negative) amplitude in the MPH session (−17.18 ± 14.33 µV/m²) than in the placebo session (−13.67 ± 13.09 µV/m²), as well as a main effect of Overlap (F(1,77) = 50.95, *p* < 0.001, η_p_² = 0.398), with a larger (i.e., more negative) amplitude in the non-overlapping condition (−18.66 ± 14.86 µV/m²) than in the overlapping condition (−12.19 ± 12.01 µV/m²). The interaction effect Substance*Overlap did not reach significance (F(1,77) = 0.58, *p* = 0.448). Bayesian statistics revealed a value of BF_10_ = 0.16 for the interaction of Substance*Overlap, indicating positive evidence for the null hypothesis relative to the alternative hypothesis.

For the RIDE-N2_C_ at the Cz in the C-cluster, a repeated measures ANOVA with the factors Substance (placebo vs. MPH) and Overlap (overlapping vs. non-overlapping) showed a main effect of Overlap (F(177) = 71.64, *p* < 0.001, η_p_² = 0.482), with a larger (i.e., more negative) amplitude in the overlapping condition (−15.06 ± 17.60 µV/m²) than in the non-overlapping condition (0.83 ± 4.30 µV/m²). No other effects were significant (all F ≤ 0.87, all *p* ≥ 0.354). Bayesian statistics revealed a value of BF_10_ = 0.08 for the interaction of Substance*Overlap, indicating positive evidence for the null hypothesis relative to the alternative hypothesis.

For the RIDE-P3 in the C-cluster, a repeated-measures ANOVA with the factors Electrode (Cz vs. Pz), Substance (placebo vs. MPH) and Overlap (overlapping vs. non-overlapping) revealed a main effect of Electrode (F(177) = 8.24, *p* = 0.005, η_p_² = 0.097): The amplitude was higher at the Cz (24.41 ± 16.82 µV/m²) than at the Pz (18.87 ± 9.54 µV/m²). There was a main effect of Substance (F(177) = 13.04, *p* < 0.001, η_p_² = 0.145), with a higher amplitude in the MPH session (23.76 ± 11.95 µV/m²) than in the placebo session (19.53 ± 11.79 µV/m²), as well as a main effect of Overlap (F(177) = 15.55, *p* < 0.001, η_p_² = 0.168), with a higher amplitude in the overlapping condition (23.37 ± 11.39 µV/m²) than in the non-overlapping condition (19.91 ± 11.33 µV/m²). The analysis also showed an interaction between Electrode and Substance (F(177) = 5.40, *p* = 0.023, η_p_² = 0.066). Post-hoc tests showed that there was a higher amplitude in the MPH session (27.89 ± 18.94 µV/m², D(78) = 0.10, *p* = 0.045) than in the placebo session (20.94 ± 19.20 µV/m², D(78) = 0.07, *p* = 0.200) at the Cz (Z = −3.39, *p* < 0.001), while there was no difference between the two sessions at the Pz (MPH: 19.62 ± 10.48 µV/m², D(78) = 0.08, *p* = 0.200; placebo: 18.11 ± 11.13 µV/m², D(78) = 0.08, *p* = 0.200; t(77) = -1.32, *p* = 0.192). Lastly, there was an interaction effect between Electrode and Overlap (F(1,77) = 4.67, *p* = 0.034, η_p_² = 0.057). Post-hoc tests revealed a higher amplitude in the overlapping condition than in the non-overlapping condition at both the Cz (overlapping: 27.12 ± 19.85 µV/m², D(78) = 0.10, *p* = 0.044; non-overlapping: 21.71 ± 16.28 µV/m², D(78) = 0.08, *p* = 0.200; Z = −3.33, *p* < 0.001) and the Pz (overlapping: 19.62 ± 9.55 µV/m², D(78) = 0.04, *p* = 0.200; non-overlapping: 18.11 ± 10.99 µV/m², D(78) = 0.15, *p* < 0.001; Z = −2.32, *p* = 0.020), with a significantly larger binding effect at the Cz (5.41 ± 13.68 µV/m², D(78) = 0.07, *p* = 0.200) than at the Pz (1.51 ± 7.75 µV/m², D(78) = 0.08, *p* = 0.200; t(77) = 2.16, *p* = 0.034). No other effects were found to be significant (all F ≤ 1.41, all *p* ≥ 0.239). Bayesian statistics revealed a value of BF_10_ = 0.23 for the interaction of Substance*Overlap, indicating positive evidence for the null hypothesis relative to the alternative hypothesis.

## Discussion

The current study examined the effects of a pharmacological modulation of the catecholaminergic system on perception-motor integration processes during response inhibition. The pharmacological effects were examined at the behavioral and the neurophysiological level. Participants were tested in a double-blind, randomized placebo-controlled cross-over design using a Go/Nogo task which systematically varied perceptual features between the Go and Nogo trials. The neurophysiological analysis specifically investigated MPH effects with respect to the stability of the representational content during perception-motor integration, assuming that MPH affects certain fractions of information that are concomitantly coded in the neurophysiological signal.

The analysis of the behavioral data in the Nogo trials revealed a decreased false alarm rate in the MPH session compared to the placebo session. This effect was more pronounced in the overlapping Nogo condition than in the non-overlapping Nogo condition—that is, it was more pronounced when perception-motor codes had to be reconfigured. The reduction of partial repetition costs indicated that event file binding and integration were decreased after the increase of catecholaminergic concentration. A similar, but much smaller, MPH effect was observed in the non-overlapping Nogo condition, which might be due to a floor effect. The neurophysiological data revealed that these effects were most likely due to modulations of the stability of the representational content in specific fractions of information coded in the neurophysiological signal. Comparing the placebo and the MPH session in an MVPA which took into consideration the stability of the decoded representational content, differences were apparent over substantial time periods with sustained generalization over time in both the non-overlapping and the overlapping condition. However, these differences were more pronounced in the overlapping condition, and here, especially in the RIDE C-cluster. The C-cluster has previously been shown to reflect codings of processes occurring in event files^15,40–42^ and the current data thus suggests that mainly the codes specific to event files are modulated in the neurophysiological signal. Thus, although the catecholaminergic system is known to exert broad effects^61^ and modulate various functional brain systems, the impact of its pharmacological modulation appears to be specific to perception-motor integration. Out of several processes jointly reflected in the neurophysiological signal, catecholamines mostly affect how stable bindings between stimuli and responses are represented. The enhancement of the stability of information in prefrontal structures has been a prominent theme of several previous studies and theoretical accounts^18–21^. The current data suggest that accounts regarding catecholaminergic effects should take into consideration that catecholamines do not generally enhance the stability of all information being processed to the same extent. Instead, catecholaminergic effects on the stability of the representational content are specific to a fraction of information coded in the neurophysiological signal.

Another intriguing effect shown by the current findings consists in the improvement of response inhibition performance after MPH administration even though the stability of the representational content increased, which renders reconfiguration of event files more difficult. Since strong bindings and a high stability of event file representations entail higher partial repetition costs^12,14^, one might have expected the intake of MPH to result in an increase rather than a decrease of the rate of false alarms, i.e., impair inhibitory control. However, the observed improved inhibitory control could be explained in terms of the theoretical framework of the Metacontrol State Model, which is closely linked to TEC^29,30^. In brief, this account assumes that the state of cognitive processes varies along a continuum between extreme flexibility and extreme stability, the latter of which is characterized by a high degree of goal focus, i.e., the task goal is shielded from task-irrelevant stimuli and content^29,30^. For instance, event files that were created previously, but are no longer relevant to the current task goal, can be conceived as irrelevant content. Similar to the present study, previous findings have indicated that a high degree of stability in cognitive control reduces partial repetition costs^31^. Further studies have shown that the cognitive control style can be affected by the catecholaminergic system^30^. Thus, the present results of the MVPA imply a change in the stability of mental content after MPH administration and the behavioral results show a reduction in partial repetition costs after MPH administration. Taken together, these findings lead to the interpretation that the stability of the goal focus and task setting is more likely to have been influenced by MPH administration than the stability of the event files, to the effect that competing event files in the overlapping Nogo trials have less influence, thereby fostering response inhibition processes.

At the neuroanatomical level, the MPH effects in the RIDE C-cluster discussed above were associated with modulations of activity in the inferior parietal cortex, the supplementary motor area and parts of the ventral stream of visual processing in the temporal cortices. The inferior parietal cortex is associated with the updating of mental representations^54,55^, which is of particular relevance in the overlapping condition when event file reconfiguration is necessary. The supplementary motor area is thought to be essential to response inhibition processes on the one hand^1^, and stimulus-response integration processes on the other hand^15,42,62^. Both processes are required in the overlapping Nogo condition. Importantly, the modulations associated with differences between the placebo and the MPH session in the overlapping condition were apparent in areas of the ventral visual stream, which are, for example, related to the perception and categorization of colors^63–65^. Since color was a main distinctive feature of Go and Nogo stimuli in the current task, this result may indicate that the maintenance of task goals is enhanced by increased catecholaminergic levels, in particular in brain areas relevant to the task performance. However, there were also differences between the placebo and the MPH session in the overlapping Nogo condition in the RIDE S-cluster, albeit clearly smaller. These differences were related to modulations of the activity in the supplementary motor area, the anterior cingulate cortex and the orbitofrontal cortex. Activity in the supplementary motor area in this stimulus-related context is thought to reflect input integration processes^66^. These are necessary in the overlapping condition when incoming stimulus features have to be integrated into a new event file, and are particularly relevant when previously formed event files cannot be used, as is the case when the focus on the task goal is enhanced by MPH administration. The orbitofrontal cortex is thought to play an important role in goal-directed behavior and behavioral adaptation to current goals^67,68^. Thus, the modulation of orbitofrontal cortex activity induced by changes in catecholamine concentration corroborates the interpretation of the MVPA findings that an increase in catecholaminergic levels might lead to a more stable representation of the task goals. Lastly, the anterior cingulate cortex is associated with the monitoring of conflicts^59,69^, which is required in the overlapping condition when competing for event files are reactivated. Intriguingly, similar to the present study, previous findings have indicated that increased anterior cingulate cortex activation after MPH administration is associated with lower error rates^70^. Interestingly, the results did not reveal any MPH-induced activity modulations in the lateral inferior prefrontal regions, although these areas have previously been associated with response inhibition^56^.

The interpretation that the MPH effects mostly affect the stability of specific representational content encoded in the neurophysiological signal is corroborated by the standard time-domain EEG analyses using RIDE-decomposed data. Replicating previous findings^11,12^, overlapping and non-overlapping conditions were associated with amplitude modulations in various time windows (P1, N2, and P3 time windows), suggesting modulations of the perceptual gating process (cf. P1)^71,72^ and response selection and inhibition processes (N2 and P3 ERP time windows)^15,32,33,41,42,73^. However, a differential MPH effect in overlapping and non-overlapping Nogo trials was only evident for the P1 ERP-component: The binding effect was only apparent in the MPH session due to a larger impact of MPH intake in the non-overlapping condition. Further, the calculated correlations between the amplitudes of the RIDE-P1 (or their differences) and the false alarm rates (or their differences) showed that both the complexity of the mental operations with the event files and the MPH administration play a role in the relationship between the RIDE-P1 and the behavior. The P1 component is suggested to reflect top-down control of gating processes, with larger amplitudes reflecting stronger inhibition of task-irrelevant input^71,72^. This inhibition is achieved by increasing the signal-to-noise ratio^74^. Dopamine is known to affect such top-down control processes by increasing the signal-to-noise ratio of sensory input^75–79^. Therefore, the administration of MPH might specifically affect the amplitude of the P1 in the non-overlapping condition: The stimulus can be unambiguously classified as a Nogo stimulus and thus other irrelevant information can be inhibited, which is further facilitated by increased catecholaminergic levels. Nevertheless, this effect was driven by the non-overlapping condition, which is not in line with the behavioral data and can therefore hardly explain the behavioural effects.

Even though the behavioral data revealed robust results (see Bayesian analysis of the interaction of Substance*Overlap), these effects were only visible on a neurophysiological level after concatenating different EEG signal processing methods (RIDE and MVPA), each depending on assumptions that are not always easy to validate. This may reflect a limitation of the study. Nevertheless, the findings as revealed by the complex methodological approach are well in line with propositions of the theoretical frameworks motivating the study.

In summary, we show that the catecholaminergic system affects sensorimotor integration during response inhibition by modulating the stability of representational content. Importantly, catecholamine levels do not affect the stability of all aspects of information being processed during sensorimotor integration to the same extent. Instead, catecholaminergic activity affects the stability of the representational content of some codes in the neurophysiological signal, as suggested by cognitive theory. Particularly fronto-parietal cortical regions are associated with the identified mechanisms. The study shows how cognitive science theory-driven pharmacology can provide insights regarding the neurobiological basis of perception-motor integration and how catecholamines affect cognitive control.

## Methods

### Participants

A sample of *N* = 96 participants was recruited to take part in the study. None of the participants reported a (past or present) neurological or psychiatric illness in a telephone screening interview. Prior to the laboratory appointment, the participants completed the Adult Self-Report for ages 18–59 (ASR)^80^ via an online-questionnaire on SoSci.de^81^ in order to screen for any signs of psychiatric difficulties. All participants had at least average IQ ( ≥ 95) as determined with the version B of the Mehrfachwahl-Wortschatz-Intelligenztest (MWT-B)^82^ completed prior to the experiment. Furthermore, the Alcohol, Smoking and Substance Involvement Screening Test (ASSIST)^83^ was conducted at the beginning of the first appointment. Taken together, 18 participants were excluded due to scores above the cut-off value in the ASR or the ASSIST, or due to technical difficulties with the EEG recording. The final sample was comprised of *N* = 78 (33 females, age range 20–30, mean age 24.1 ± 2.8 years). All participants gave their informed written consent to participate and were either financially reimbursed or received course credit for their participation. The study was conducted with approval from the ethics committee of the Faculty of Medicine of the TU Dresden.

### MPH administration

In a randomized, double-blind cross-over design, the participation in the study entailed taking part in two sessions. The time period between the two appointments consisted of a minimum of 24 h up to a maximum of 14 days (mean interval 5.1 days ± 3.4). There were two groups that the participants were randomly assigned to: In the first group, the MPH was administered during the first session and an indistinguishable placebo during the second session (MPH-first group, *n* = 39). In the second group, the placebo was given during the first session and the MPH during the second session (placebo-first group, *n* = 39). The groups did not differ regarding age (*p* = 0.683), gender distribution (*p* = 0.819), IQ (*p* = 0.451) or days between appointments (*p* = 0.646). Accordingly, the study design also allowed for the investigation of the interaction of the effects of prior task familiarization and the impact of MPH. The order of substance administration was unknown to both the participants and the researchers. In line with prior studies by our group examining the interplay of MPH and learning effects^84,85^, a single dose of immediate-release MPH (0.25 mg/kg body weight) was administered to the participants in the MPH session. In order to ensure task completion during the time period of the peak of MPH plasma levels^86,87^, the experimental testing commenced approximately 75 min after substance administration.

### Task

In order to investigate the effects of MPH on event file coding in the context of response inhibition, the TEC Go/Nogo task established by Chmielewski and Beste^12^ was administered to the participants. The task was shown to the participants on a 17-inch CRT computer screen which was placed approx. at a distance of 60 cm in front of the participants. Prior to the start of the experiment, the task instructions were presented to the participants in both written and verbal form. Subsequently, the participants established a task familiarization by carrying out 30 trials in a training session. Importantly, the training trials did not allow for a long-term learning effect with regard to the event files, since these are established automatically.

During the intertrial interval, which was jittered between 700 and 1100 ms, a white fixation cross was presented at the center of the screen. In every trial, a stimulus was shown to the participants for 450 ms. The trial was either concluded after 1700 ms or upon the response given by the participant. The task consisted in Go trials and Nogo trials which both could either be overlapping or non-overlapping. Altogether, there were one non-overlapping condition and two overlapping conditions for both the Go trials and the Nogo trials, respectively. In order to prompt a prepotent response tendency, the ratio of the number of Go trials and Nogo trials was 7:3 (196 trials for each Go condition, 84 trials for each Nogo condition). The entire task consisted in seven blocks of equal length in which all types of trials occurred pseudorandomized and with equal frequency, thereby constituting a counterbalanced presentation of the stimuli.

The participants were asked to respond in Go trials by pressing the space key and to withhold any response during Nogo trials. The presentation of the word “PRESS” in the color green in Go trials and the word “STOPP” in the color red in Nogo trials resulted in the respective non-overlapping trials. The overlapping Go trials were established by the presentation of the word “DRÜCK” (German for “press”) in the color white or the five letters “XXXXX” in the color blue. In the overlapping Nogo trials, either the word “DRÜCK” in the color blue or the five letters “XXXXX” in the color white were presented. Thus, there was a substantial overlap between the features (i.e., colors and letters) of these Go trials and Nogo trials. The inclusion of stimulus features that are also included in Go trials, in these Nogo trials required the reconfiguration of the established event file, thereby resulting in a higher false alarm rate in the Nogo trials. For all conducted analyses, the effect of the stimulus feature overlap was examined by subtracting the non-overlapping condition from the overlapping condition, which is referred to as a binding effect.

### EEG recording and pre-processing

Using a BrainAmp Amplifier and the Brain vision Recorder 1.2 software (Brain Products, Germany), the EEG data were recorded from 60 Ag/AgCl electrodes arranged in an equidistant setup at a sampling rate of 500 Hz. The ground electrode was located at the coordinates θ = 58, ϕ = 78, while the reference electrode was placed at the coordinates θ = 90, ϕ = 90. The Brain Vision Analyzer 2 software package (Brain Products Inc., Germany) was used to perform the off-line preprocessing. After re-sampling the data to 256 Hz, a bandpass filter was applied (IIR filter: 0.5–40 Hz with an order of 8 and a notch filter at 50 Hz). Channels without any activity were eliminated and then the data was re-referenced to an average reference. In a manual inspection, technical artifacts were discarded and the following independent component analysis (ICA, Infomax) removed periodic artifacts. Residual artifacts were removed in a second raw data inspection. Then, a topographic interpolation of the previously discarded channels was performed. Individual segments locked to the respective stimulus of each trial were established (−2000 to 2000 ms) for all conditions. All segments that showed amplitudes exceeding 200 µV or lower than −200 µV, or displaying activity that was lower than 0.5 µV for more than 100 ms, were discarded in an automatic artifact rejection. Current source density (CSD) transformation was applied for a reference-free evaluation of the EEG data^88^. Finally, a baseline correction was carried out which was based on the mean activity in the time window from −200 ms to 0 ms prior to stimulus onset.

### Temporal EEG signal decomposition

Residue iteration decomposition (RIDE) was used to further process the baseline-corrected EEG data. This temporal decomposition method is based on the assumption that event-related potentials (ERPs) consist of different components with variable delays that can be associated with different stages of cognitive processing^45^. RIDE iteratively decomposes ERPs at the level of a single trial into components (so-called clusters), with static and variable latency based on their timing and temporal variability^45^. Thereby, after decomposition, each derived cluster has the same data structure as the undecomposed EEG data, i.e., the same number of channels and data points^44^. Because RIDE separates component clusters only by their latency variability, not by their scalp distributions and waveforms^45^, it is not critical to use CSDs. In the presence of stimulus and response time points, RIDE derives a stimulus-associated S-cluster and a response-associated R-cluster, respectively, based on these time points. Because only Nogo trials are evaluated for the present paradigm, there is no time marker for the response in the current analysis and thus no derivation of an R cluster^47^. The time marker for the S-cluster, i.e., latency (S_L_), is set to the time of stimulus onset in the EEG. The time markers for deriving the C-cluster (C_L_) are iteratively estimated and improved. To estimate S, C is subtracted from each trial. Then, the residual of all trials is aligned with the latency S_L_, which results in S as the median of the waveform for all time points. Conversely, S is subtracted from each trial to determine C, followed by the same procedure described above. RIDE decomposition was performed separately for each electrode channel according to established procedures^43^ using the RIDE toolbox (manual available at http://cns.hkbu.edu.hk/RIDE.htm). A time window function was used to extract the waveform of each RIDE component. In accordance with the time windows of classical ERP components in response inhibition and similar to a previous study on this paradigm^11^, the time window for the S-cluster was set from −200 to 600 ms relative to stimulus onset. For the C-cluster, the time window was set from 150 to 800 ms relative to stimulus onset.

Based on existing literature^11,89–92^, the RIDE components of interest were selected and their typical time windows and topography were used to search for these components in the current data. Taking this information into account, the specific time windows for quantifying the RIDE components in the current study were determined based on visual inspection of the grand averages. The quantification itself was then performed at the single subject level. In the S-cluster, the RIDE-P1 was quantified from 95 to 115 ms relative to stimulus onset and the RIDE-N1 was quantified from 150 to 180 ms relative to stimulus onset, both at electrodes P7 and P8. Moreover, the RIDE-N2_S_ (i.e., the N2 in the S-cluster) was quantified from 240 to 290 ms relative to stimulus onset at electrode Cz. In the C-cluster, the RIDE-N2_C_ (i.e., the N2 in the C-cluster) was quantified at electrode Cz from 235 to 265 ms in the non-overlapping condition and from 335 to 365 ms in the overlapping condition. The RIDE-P3 was quantified at electrode Cz from 370 to 420 ms in the non-overlapping condition and from 460 to 510 ms in the overlapping condition as well as at electrode Pz from 410 to 460 ms in the non-overlapping condition and from 480 to 560 ms in the overlapping condition.

### Multivariate pattern analysis (MVPA)

In order to differentiate between the placebo and the MPH session in both the non-overlapping and the overlapping condition, a multivariate pattern analysis (MVPA) was applied to the undecomposed EEG data as well as to the RIDE-decomposed EEG data using the MVPA-light toolbox^38^. In the present study, a binary classification across time was performed to identify time points showing different values between the placebo and the MPH session. To further characterize the temporal dynamics of the representational content, a temporal generalization analysis was conducted. In order to balance the number of trials in the placebo and the MPH session and thereby avoid an overfitting problem during MVPA, the number of trials of each participant was undersampled. The undersampling was performed using the embedded MVPA-light algorithm during MVPA for the undecomposed EEG data, and prior to MVPA for the RIDE clusters to ensure that the selected samples were identical for all RIDE clusters. Only signals from 0 to 1500 ms relative to stimulus presentation were fed into the MVPA for each trial. The analyses regarding the binary classification and the temporal generalization were conducted separately for each participant and the undecomposed data/RIDE clusters using the same parameter settings.

The contrast between the placebo and the MPH session was computed using a two-class L1-Support Vector Machine (SVM) classifier. This classifier was chosen because of its robustness regarding outliers. The SVM classifier further performs better for strongly non-Gaussian or noisy data than the default linear discriminant analysis (LDA) classifier^38^. For the computations, a cross-validation method with five folds was applied. All other parameter settings were left unchanged compared to the default settings of the MVPA-light toolbox. Significant classification performance was represented by the area under the ROC curve (AUC) and identified using cluster-based permutation tests. For the binary classification across time and the temporal generalization, the cluster-based permutation tests were based on the non-parametric Wilcoxon tests on each time point using AUC values. A chance level of 0.5 was the null value for AUC. The reference distribution of the permutation test was computed with 1000 random draws. The threshold for the Wilcoxon tests was set at *p* = 0.05 in accordance with previous studies by our group that used a MVPA to examine event file dynamics^11,15,93–95^. The cluster-level statistical values were computed as the sum of all Wilcoxon-values within time points.

### Source localization analysis (sLORETA)

Standard low resolution brain electromagnetic tomography (sLORETA)^48^ was used for the localization of sources of modulations in the overlapping Nogo condition for the time periods in which the classification accuracy in the MVPA was above-chance. The sLORETA was only performed in the overlapping condition since event file reconfiguration processes only in this condition and the effects of substance administration on the behavior were strongest in this condition (see results section for details). It has been shown in previous studies^96–98^ that sources can be reliably estimated by this software. Moreover, sLORETA also provides a unique solution to the inverse problem and gives results without a localization bias^98,99^. The intracerebral volume is divided into 6,239 voxels at 5 mm spatial resolution for the analyses. Based on the MNI152 template^100^, the standardized current density is calculated in a realistic head model at each voxel^101^. The voxel-based sLORETA images of the placebo and the MPH session were contrasted, using sLORETA built-in voxel-wise randomization tests with 5,000 permutations on the basis of statistical nonparametric mapping (SnPM). Voxels with significant differences between the compared sessions were then localized in the MNI brain (*P* < 0.05, corrected for multiple comparisons).

### Statistics and reproducibility

For the analysis of the behavioral data, i.e., the Nogo false alarm rates, a mixed-effects ANOVA with the within-subject factors Overlap (non-overlapping vs. overlapping condition) and Substance (placebo vs. MPH session) and the between-subjects factor Group (Placebo-first vs. MPH-first) was conducted. Since the factor Group did not affect the behavioral performance (refer to the Results section for details), it was not included in the analysis of the RIDE time domain data. For the RIDE time domain data, repeated-measures ANOVAs with the within-subject factors Overlap (non-overlapping vs. overlapping condition), Substance (placebo vs. MPH session) and, when necessary, the within-subject factor Electrode were conducted. Prior to any post-hoc tests, the normal distribution of the variables was examined using a Kolmogorov-Smirnov test. Post-hoc tests were then conducted using paired-sample t-tests or Wilcoxon tests, respectively. Means and standard deviations are reported for the descriptive statistics. Since the interaction of the factors Overlap and Substance is of particular interest, the results concerning this interaction were validated using Bayesian statistics. In order to do so, the template provided by Masson^102^ based on the suggestions by Wagenmakers^103^ was used to calculate the Bayes factor BF_10_, which denotes the probability of the alternative hypothesis relative to the null hypothesis. The classification of the results was done according to Raftery^104^. Values of BF10 < 1 reflect evidence for the null hypothesis relative to the alternative hypothesis (weak evidence: 0.33–1; positive evidence: 0.05–0.33; strong evidence: 0.01–0.05; very strong evidence: <0.01), whereas values of BF10 > 1 reflect evidence for the alternative hypothesis relative to the null hypothesis (weak evidence: 1–3; positive evidence: 3–20; strong evidence: 20–150; very strong evidence: >150)^104^. For any RIDE components that showed the interaction of interest of Overlap*Substance, an additional correlation analysis of the amplitudes in the four conditions (and amplitude differences, i.e., binding effect and substance effect) with the corresponding false alarm rates in the behavioral data was performed.

The sample size is larger compared to previous studies using MVPA on RIDE-decomposed EEG data^11,94^ and the sample size is larger than comparable previous work using the same substance (i.e., MPH)^85^. Details regarding the statistics for the MVPA are given in the respective part of the methods section (see above). All data is available in OSF.

### Reporting summary

Further information on research design is available in the Nature Research Reporting Summary linked to this article.

## Supplementary information

Supplementary Information

Description of Additional Supplementary Files

Supplementary Data 1

reporting summary

## Data Availability

Data can be downloaded from https://osf.io/vepb6/ (DOI 10.17605/OSF.IO/VEPB6). Source data for Fig. 1 and Supplementary Figs. 1 and 2 have been provided in Supplementary Data 1 - The source data behind Fig. 1, Supplementary Figs. 1 and 2.

## Code availability

Further custom code used to process the data can be found here: https://osf.io/vepb6/ (10.17605/OSF.IO/VEPB6).
