# Supplementary material for: Cognitive science theory-driven pharmacology elucidates the neurobiological basis of perception-motor integration
Source: Commun Biol. 2022 Sep 6;5:919. doi: 10.1038/s42003-022-03864-1 (PMC9448745; doi:10.1038/s42003-022-03864-1)
Supplement: Supplementary file 1 — Supplementary Information [file 42003_2022_3864_MOESM1_ESM.pdf]

Supplementary Information

Cognitive science theory-driven pharmacology elucidates the neurobiological basis of perception-motor integration

Elena Eggert, Astrid Prochnow, Veit Roessner, Christian Frings, Alexander Münchau, Moritz Mückschel, Christian Beste

Supplementary Note 1: Behavioral analyses of Go trials

Go hit rate

Supplementary Figure 1 displays the distribution of the Go hit rates separately for each group (figure part a) and an illustration of the interaction effect (figure part b). The mixed-effects ANOVA of the Go hit rate revealed a main effect of the factor Overlap ($F(1,76) = 37.79$, $p < .001$, $\eta_p^2 = .332$), with higher hit rates in the non-overlapping (.993 ± .012) than in the overlapping condition (.988 ± .015). Further, the analysis revealed a significant interaction of Substance*Overlap*Group ($F(1,76) = 5.99$, $p = .017$, $\eta_p^2 = .073$). In the Placebo-first group, there was a significant main effect of Overlap ($F(1,38) = 19.26$, $p < .001$, $\eta_p^2 = .336$) with higher hit rates in the non-overlapping (.992 ± .015) than in the overlapping condition (.986 ± .019), and a significant interaction of Substance*Overlap ($F(1,38) = 8.05$, $p = .007$, $\eta_p^2 = .175$). Prior to post-hoc tests, Kolmogorov-Smirnov-tests were conducted for all post-hoc variables to test for normality. None of the variables was normally distributed ($D(78) \geq .20$, $p < .001$). Wilcoxon-tests revealed significant binding effects in the placebo session (non-overlapping: .990 ± .013; overlapping: .982 ± .019; $Z = -3.85$, $p < .001$) as well as in the MPH session (non-overlapping: .993 ± .023; overlapping: .989 ± .025; $Z = -3.26$, $p = .001$), with a larger binding effect in the placebo session than in the MPH session ($Z = -2.54$, $p = .011$). The effect of Substance was larger in the overlapping than in the non-overlapping condition ($Z = -2.54$, $p = .011$). However, in the MPH-first group, only the main effect of Overlap was significant ($F(1,38) = 19.48$, $p < .001$, $\eta_p^2 = .339$), but the main effect of Substance and the interaction of the factors did not reach significance ($F \leq .30$, $p \geq .589$).

Go reaction times

The distribution of the Go reaction times (RTs) separately for each group and an illustration of the interaction effect is displayed in Supplementary Figure 2. The mixed-effects ANOVA of the Go RTs revealed a main effect of the factor Overlap ($F(1,76) = 25.93$, $p < .001$, $\eta_p^2 = .254$), with faster RTs in the overlapping (449 ± 51 ms) than in the non-overlapping condition (438 ± 52 ms). Further, the analysis revealed significant interactions of Substance*Group ($F(1,76) = 49.42$, $p < .001$, $\eta_p^2 = .394$) and Substance*Overlap*Group ($F(1,76) = 4.64$, $p = .042$, $\eta_p^2 = .053$). Prior to post-hoc tests regarding the three-way interaction, Kolmogorov-Smirnov-tests were conducted for all post-hoc variables to test for normality. The interaction of Substance*Group was existent in the non-overlapping ($F(1,76) = 55.92$, $p < .001$, $\eta_p^2 = .424$) as well as in the overlapping condition ($F(1,76) = 38.88$, $p < .001$, $\eta_p^2 = .338$). In the non-overlapping condition, the groups differed in the placebo session (Placebo-first: 462 ± 63 ms, $D(78) = .11$, $p = .200$; MPH-first: 419 ± 38 ms, $D(78) = .11$, $p = .200$; $t(62.17) = 3.64$, $p < .001$) as well as in the MPH session (Placebo-first: 429 ± 62 ms, $D(78) = .25$, $p < .001$; MPH-first: 442 ± 49 ms, $D(78) = .13$, $p = .079$; $Z = -1.97$, $p = .048$), whereas in the overlapping condition, the groups differed only in the placebo session (Placebo-first: 473 ± 61 ms, $D(78) = .14$, $p = .49$; MPH-first: 429 ± 43 ms, $D(78) = .09$, $p = .200$; $Z = -3.15$, $p =.002$), but not in the MPH session (Placebo-first: 444 ± 57 ms, $D(78) = .18$, $p = .002$; MPH-first: 449 ± 51 ms, $D(78) = .12$, $p = .180$; $Z = -.86$, $p = .387$).

Supplementary Figure 1: Hit rates of the non-overlapping and overlapping Nogo conditions in the placebo and MPH session

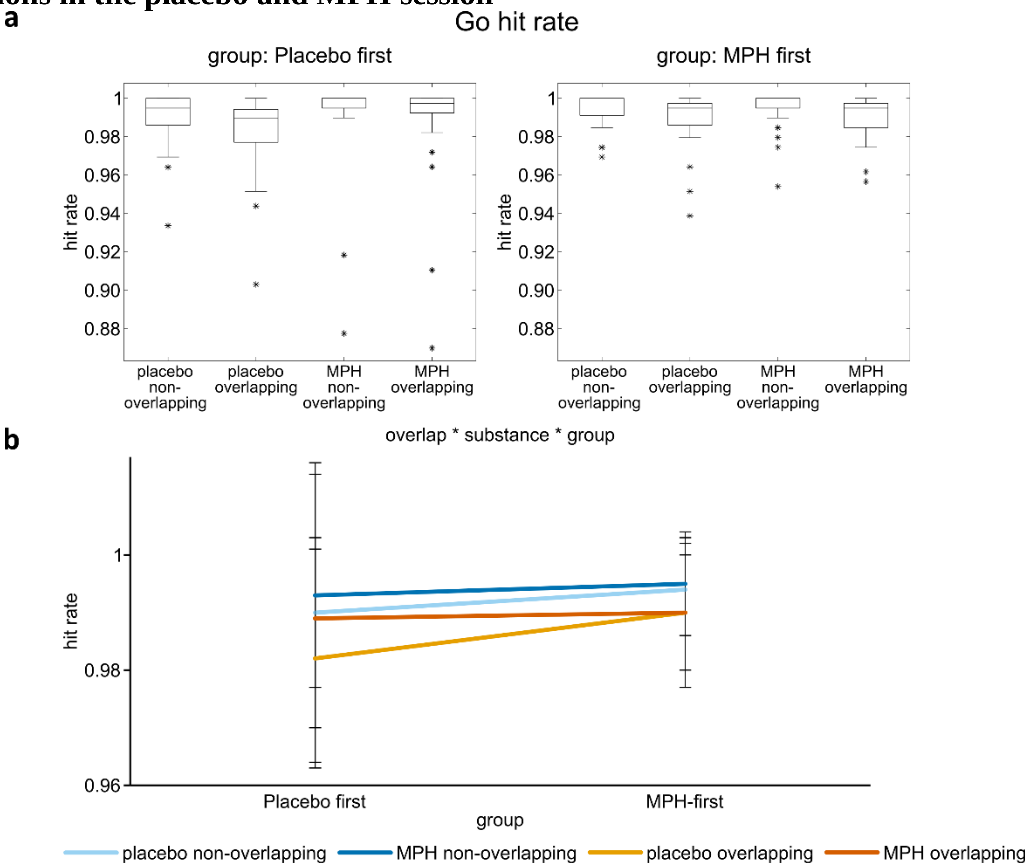

(a) Boxplots of the distribution of the hit rates of the non-overlapping and overlapping Go conditions in the placebo and MPH sessions separately for the Placebo-first group and the MPH-first group. The sample median is shown as the line inside the box, the lower and upper quartile are shown as the bottom and top edges of the box, the ends of the whiskers denote the non-outlier minimum and maximum, respectively, asterisks denote outliers. (b) Illustration of the interaction of the factors Overlap, Substance and Group (same data as figure part (a)). Error bars represent standard deviations.

Supplementary Figure 2: Reaction times of non-overlapping and overlapping Nogo conditions in the placebo and MPH session

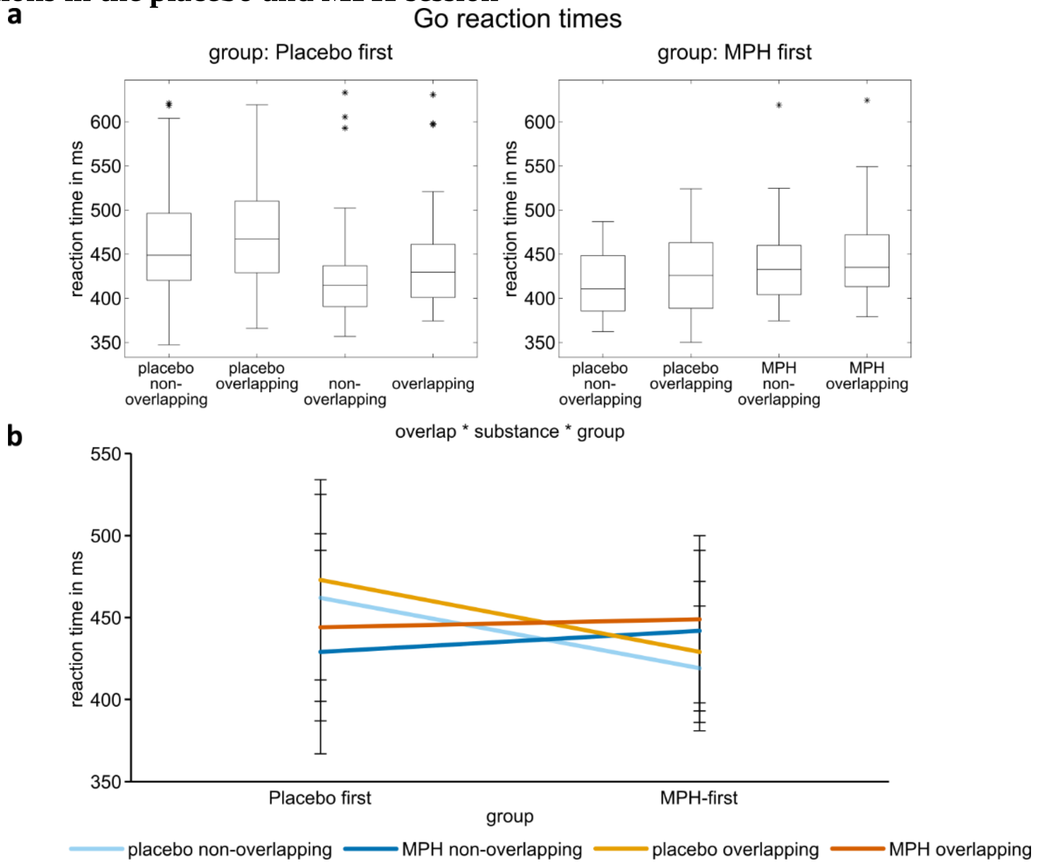

(a) Boxplots of the distribution of the reaction times of the non-overlapping and overlapping Go conditions in the placebo and MPH sessions separately for the Placebo-first group and the MPH-first group). The sample median is shown as the line inside the box, the lower and upper quartile are shown as the bottom and top edges of the box, the ends of the whiskers denote the non-outlier minimum and maximum, respectively, asterisks denote outliers. (b) Illustration of the interaction of the factors Overlap, Substance and Group (same data as figure part (a)). Error bars represent standard deviations.

Supplementary Note 2: Multivariate pattern analyses of the binding effects in placebo and MPH

The MVPA was applied to the undecomposed as well as to the RIDE-decomposed data in the time window of 0 to 1500 ms relative to the stimulus onset. The classes of non-overlapping and overlapping trials were compared in the placebo session as well as in the MPH session. In order to examine the stability of the representations, temporal generalization matrices were computed. Supplementary Figure 3 displays the results of the binary classification in the overlapping and the non-overlapping condition (figure part a) as well as the results of the temporal generalization MVPA in the non-overlapping (figure part b) and the overlapping condition (figure part c).

In the undecomposed data, the analysis revealed significant differences ($p < .05$) between the placebo and MPH sessions in both the overlapping and the non-overlapping condition. In the placebo session, an above-chance classification performance with a classification accuracy ranging between .51 and .67 (mean classification accuracy: .57) was observed in the time period from 94 to 1,090 ms after stimulus onset with short breaks from 965 to 973 ms and 996 to 1008 ms after stimulus onset. The temporal generalization around the diagonal had a duration of about 290 ms on average. In the MPH session, an above-chance classification performance with a classification accuracy ranging between .51 and .67 (mean classification accuracy: .58) was observed in the time period from 94 to 1012 ms after stimulus onset with a short break from 945 to 953 ms after stimulus onset. The temporal generalization around the diagonal had a duration of about 270 ms on average. As can be seen, the range and duration of above-chance classification accuracy did not differ substantially.

In the RIDE S-cluster, the analysis revealed significant differences ($p < .05$) between the placebo and MPH sessions in both the overlapping and the non-overlapping condition. With a classification accuracy of .51 to .85 (mean classification accuracy: .70), an above-chance classification performance was revealed in the time window of 86 to 559 ms in the placebo session. The temporal generalization around the diagonal had a duration of about 350 ms on average. In the MPH session, an above-chance classification performance with a classification accuracy of .51 to .88 (mean classification accuracy: .73) was shown in the time window of 66 to 547. The temporal generalization around the diagonal had a duration of about 315 ms on average. As can be seen, the range and duration of above-chance classification accuracy did not differ substantially.

Similarly, the analysis of the RIDE C-cluster data showed significant differences ($p < .05$) for both the overlapping condition and the non-overlapping condition between placebo and MPH sessions. In the placebo session, an above-chance classification performance with a classification accuracy ranging between .55 and .88 (mean classification accuracy: .79) was observed in the time period from 195 to 520 ms after stimulus onset. The temporal generalization around the diagonal had a duration of about 340 ms on average. In the MPH session, an above-chance classification performance with a classification accuracy ranging between .53 and .92 (mean classification accuracy: .82) was observed in the time period from 191 to 539 ms after stimulus onset. The temporal generalization around the diagonal had a duration of about 320 ms on average. As can be seen, the range and duration of above-chance classification accuracy did not differ substantially.

Taken together, the findings demonstrate a successful classification performance for the undecomposed data as well as for the S-cluster and the C-cluster.

Supplementary Figure 3: Results of the MVPA comparing the non-overlapping condition and the overlapping condition in Nogo trials

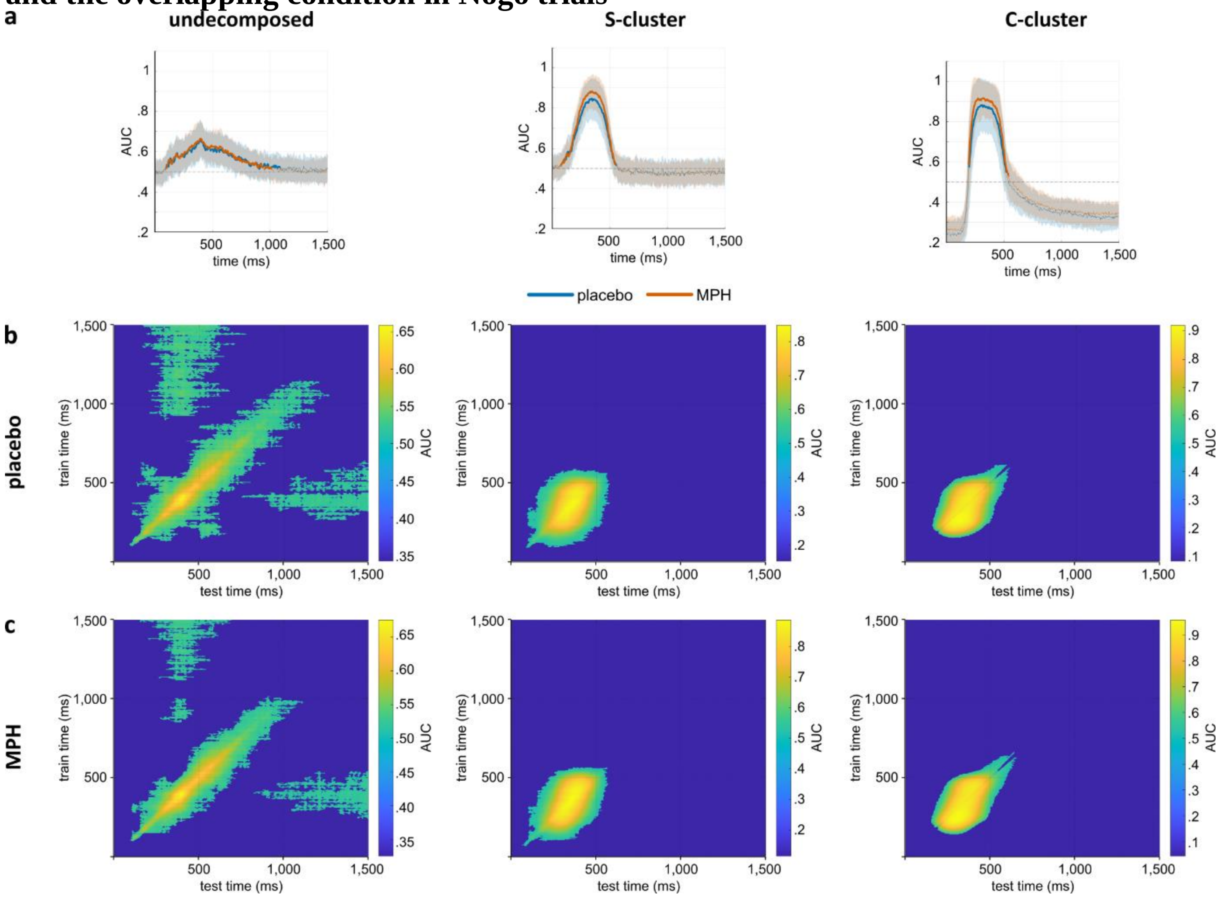

Results of the MVPA comparing the non-overlapping condition and the overlapping condition in Nogo trials in the undecomposed EEG data, in the RIDE S-cluster and in the RIDE C-cluster. Figure part (a) shows the AUC for the diagonal activity in the placebo session (blue line) and in the MPH session (orange line); thick lines indicate significant above-chance classification, the shading around the line represents the standard deviation of the AUC across the sample. Figure part (b) shows the temporal generalization matrices for the placebo session, figure part (c) shows the temporal generalization matrices for the MPH session; color indicates AUC.

Supplementay Figure 4: Results of the MVPA comparing the placebo session and the MPH session in Nogo trials with alpha = .001

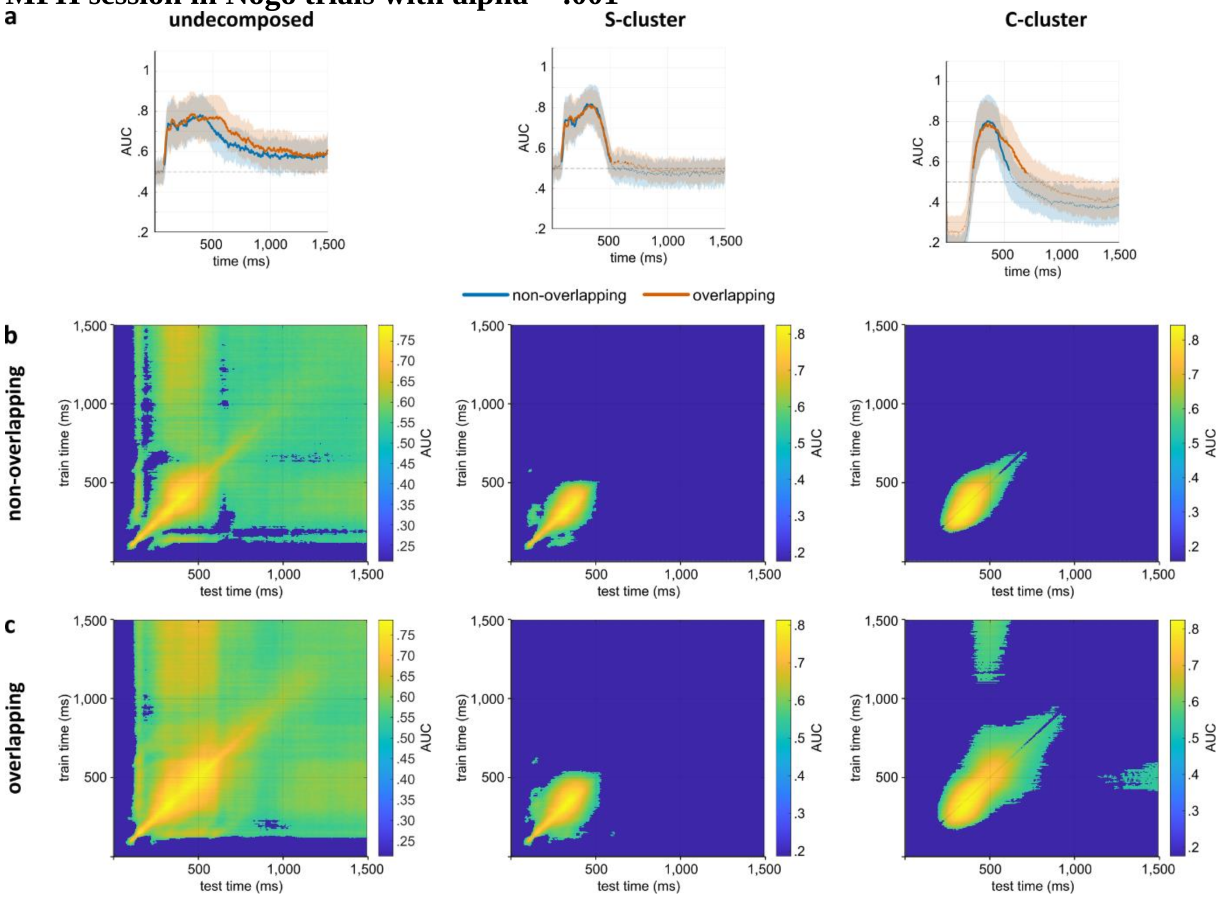

Results of the MVPA (alpha = .001) comparing the placebo session and the MPH session in Nogo trials in the undecomposed EEG data, in the RIDE S-cluster and in the RIDE C-cluster. Figure part (a) shows the AUC for the diagonal activity in the non-overlapping condition (blue line) and in the overlapping condition (orange line); thick lines indicate significant above-chance classification, the shading around the line represents the standard deviation of the AUC across the sample. Figure part (b) shows the temporal generalization matrices for the non-overlapping condition, figure part (c) shows the temporal generalization matrices for the overlapping condition; color indicates AUC.

Supplementay Figure 5: Results of the MVPA comparing the non-overlapping condition and the overlapping condition in Nogo trials with alpha = .001

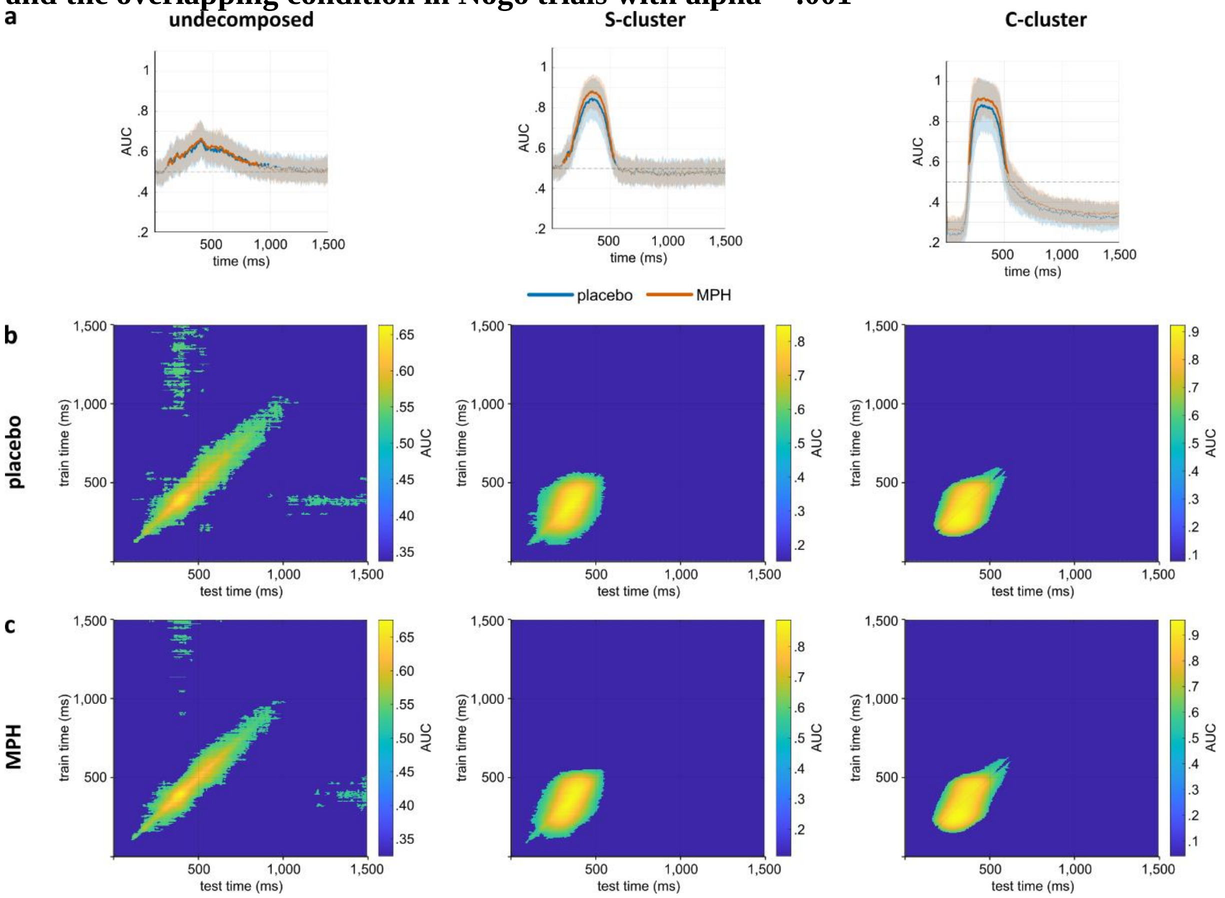

Results of the MVPA (alpha = .001) comparing the non-overlapping condition and the overlapping condition in Nogo trials in the undecomposed EEG data, in the RIDE S-cluster and in the RIDE C-cluster. Figure part (a) shows the AUC for the diagonal activity in the placebo session (blue line) and in the MPH session (orange line); thick lines indicate significant above-chance classification, the shading around the line represents the standard deviation of the AUC across the sample. Figure part (b) shows the temporal generalization matrices for the placebo session, figure part (c) shows the temporal generalization matrices for the MPH session; color indicates AUC.
