# Supplementary material for: Cognitive science theory-driven pharmacology elucidates the neurobiological basis of perception-motor integration
Source: Commun Biol. 2022 Sep 6;5:919. doi: 10.1038/s42003-022-03864-1 (PMC9448745; doi:10.1038/s42003-022-03864-1)
Supplement: Supplementary file 2 — Description of Additional Supplementary Files [file 42003_2022_3864_MOESM2_ESM.pdf]

Description of Additional Supplementary Files

File name: Supplementary Data 1
Description: The source data behind Figure 1, Supplementary Figure 1, and Supplementary Figure 2
