# Supplementary material for: Cognitive science theory-driven pharmacology elucidates the neurobiological basis of perception-motor integration
Source: Commun Biol. 2022 Sep 6;5:919. doi: 10.1038/s42003-022-03864-1 (PMC9448745; doi:10.1038/s42003-022-03864-1)
Supplement: Supplementary file 4 — reporting summary [file 42003_2022_3864_MOESM4_ESM.pdf]

nature portfolio

Corresponding author(s): Christian Beste

Last updated by author(s): Jan 13, 2021

# Reporting Summary

Nature Portfolio wishes to improve the reproducibility of the work that we publish. This form provides structure for consistency and transparency in reporting. For further information on Nature Portfolio policies, see our Editorial Policies and the Editorial Policy Checklist.

## Statistics

For all statistical analyses, confirm that the following items are present in the figure legend, table legend, main text, or Methods section.

| n/a | Confirmed | |
|---|---|---|
| ☐ | ☒ | The exact sample size (*n*) for each experimental group/condition, given as a discrete number and unit of measurement |
| ☐ | ☒ | A statement on whether measurements were taken from distinct samples or whether the same sample was measured repeatedly |
| ☒ | ☐ | The statistical test(s) used AND whether they are one- or two-sided<br>*Only common tests should be described solely by name; describe more complex techniques in the Methods section.* |
| ☒ | ☐ | A description of all covariates tested |
| ☐ | ☒ | A description of any assumptions or corrections, such as tests of normality and adjustment for multiple comparisons |
| ☐ | ☒ | A full description of the statistical parameters including central tendency (e.g. means) or other basic estimates (e.g. regression coefficient) AND variation (e.g. standard deviation) or associated estimates of uncertainty (e.g. confidence intervals) |
| ☒ | ☐ | For null hypothesis testing, the test statistic (e.g. *F*, *t*, *r*) with confidence intervals, effect sizes, degrees of freedom and *P* value noted<br>*Give P values as exact values whenever suitable.* |
| ☒ | ☐ | For Bayesian analysis, information on the choice of priors and Markov chain Monte Carlo settings |
| ☒ | ☐ | For hierarchical and complex designs, identification of the appropriate level for tests and full reporting of outcomes |
| ☐ | ☒ | Estimates of effect sizes (e.g. Cohen's *d*, Pearson's *r*), indicating how they were calculated |

*Our web collection on statistics for biologists contains articles on many of the points above.*

## Software and code

Policy information about availability of computer code

| | |
|---|---|
| Data collection | EEG recording: BrainVision Recorder (Brain Products GmbH)<br>Stimulus Presentation: Presentation 14.9 (Neurobehavioral Systems, Inc.) |
| Data analysis | Statistics behavioral data: IBM SPSS Statistics 27 (IBM)<br>EEG processing: BrainVision Analyzer 2 (Brain Products GmbH); Matlab (The MathWorks, Inc.), MVPA light toolbox (Treder, 2020)<br>Bayesian analyses: JASP (version 0.16)<br>Source localization: sLORETA (Pascual-Marqui, KEY Institute for Brain-Mind Research, University Hospital of Psychiatry Zurich; http://www.unizh.ch/keyinst/NewLORETA/sLORETA/sLORETA.htm) |

For manuscripts utilizing custom algorithms or software that are central to the research but not yet described in published literature, software must be made available to editors and reviewers. We strongly encourage code deposition in a community repository (e.g. GitHub). See the Nature Portfolio guidelines for submitting code & software for further information.

## Data

Policy information about availability of data

All manuscripts must include a data availability statement. This statement should provide the following information, where applicable:

- Accession codes, unique identifiers, or web links for publicly available datasets
- A description of any restrictions on data availability
- For clinical datasets or third party data, please ensure that the statement adheres to our policy

Data can be downloaded from https://osf.io/vepb6/

# Field-specific reporting

Please select the one below that is the best fit for your research. If you are not sure, read the appropriate sections before making your selection.

☐ Life sciences ☒ Behavioural & social sciences ☐ Ecological, evolutionary & environmental sciences

For a reference copy of the document with all sections, see nature.com/documents/nr-reporting-summary-flat.pdf

# Behavioural & social sciences study design

All studies must disclose on these points even when the disclosure is negative.

| | |
|---|---|
| Study description | Quantitative, mixed design of experimental conditions |
| Research sample | Healthy participants in the age range of 20 to 30 years |
| Sampling strategy | Convenience sample |
| Data collection | EEG data and behavioral data (computer keyboard responses during an experiment) |
| Timing | April 2019 until June 2021 |
| Data exclusions | 18 participants were excluded due to scores above the cut-off value in the Adult Self-Report for ages 18-59 (ASR) or the Alcohol, Smoking and Substance Involvement Screening Test (ASSIST), or due to technical difficulties with the EEG recording |
| Non-participation | There were no drop-outs |
| Randomization | Randomized, double-blind cross-over study design regarding substance administration (MPH/placebo) |

# Reporting for specific materials, systems and methods

We require information from authors about some types of materials, experimental systems and methods used in many studies. Here, indicate whether each material, system or method listed is relevant to your study. If you are not sure if a list item applies to your research, read the appropriate section before selecting a response.

### Materials & experimental systems

| n/a | Involved in the study | |
|---|---|---|
| ☒ | ☐ | Antibodies |
| ☒ | ☐ | Eukaryotic cell lines |
| ☒ | ☐ | Palaeontology and archaeology |
| ☒ | ☐ | Animals and other organisms |
| ☐ | ☒ | Human research participants |
| ☒ | ☐ | Clinical data |
| ☒ | ☐ | Dual use research of concern |

### Methods

| n/a | Involved in the study | |
|---|---|---|
| ☒ | ☐ | ChIP-seq |
| ☒ | ☐ | Flow cytometry |
| ☒ | ☐ | MRI-based neuroimaging |

## Human research participants

Policy information about studies involving human research participants

| | |
|---|---|
| Population characteristics | The final sample was comprised of N=78 (33 females, age range 20-30, mean age 24.1 ± 2.8 years). None of the participants reported a (past or present) neurological or psychiatric illness in a telephone screening interview. All participants had at least |

average IQ (≥ 95). All participants gave their written consent to participate and were either financially reimbursed or received course credit for their participation.

| | |
|---|---|
| Recruitment | Convenience sample, participants were recruited via online study advertisements or contacted based on an in-house recruitment database |
| Ethics oversight | IRB of the TU Dresden |

Note that full information on the approval of the study protocol must also be provided in the manuscript.
